# Incidence of bacterial meningitis in South East Asia region

**DOI:** 10.1186/1471-2458-12-S2-A30

**Published:** 2012-11-27

**Authors:** Nimetcan Mehmet Orhun, Zaleha Md Isa, Azam Rahimi, Isidore Koffi Kouadio, Hasanain Faisal Ghazi, Syed Mohamed Aljunid

**Affiliations:** 1 United Nations University-International Institute for Global Health, Universiti Kebangsaan Malaysia Medical Centre, Jalan Yaacob Latiff, 56000 Kuala Lumpur, Malaysia; 2 Department of Community Health, Universiti Kebangsaan Malaysia Medical Centre, Jalan Yaacob Latiff, 56000 Kuala Lumpur, Malaysia

## Background

Acute bacterial meningitis (BM) constitutes a significant global public health problem. Worldwide, it has been estimated that 1—2 million cases of BM occur annually. The problem is more significant in resource-poor countries including those in some regions of Sub-Saharan Africa, Southeast Asia, and Latin America. Aim of study was to measure Incidence of BM in Southeast Asia countries based on published data.

## Materials and methods

An extensive review was carried out on BM from 2000 to 2011 among 11 countries (Bangladesh, Bhutan, North Korea, India, Indonesia, Maldives, Myanmar, Nepal, Sri Lanka, Thailand and Timor- Leste) in WHO South- East Asian region. Three individual’s researchers independently screened the titles and abstracts of each citation retrieved from the search term and identified the articles for full review. Literature searches were conducted using the PubMed database, Google scholar, The Lancet, and each countries ministry of Health website and were limited to articles written in English. The search term combinations used to search the knowledgebase included BM, Incidence rate, epidemiology and clinical burden.

## Results

While there were published data on BM in Bangladesh, India, Indonesia, Nepal, Sir Lanka and Thailand, such data or report were not available for other countries. After the application of the inclusion, exclusion and quality criteria, the comprehensive review of the literature yielded 8 articles with data for BM. The incidence of BM varied from country to country, ranging from 18.3 to 24.6 /100,000 populations. Its incidence was highest in Thailand, and lowest in India.

## Conclusions

In spite of the scarcity of published data on incidence of bacterial meningitis in the South East Asian countries, this review suggests that it may be high and more efforts are recommended for developing practical mechanisms in reporting of this condition.

